# Cultural competence of Australian dental students

**DOI:** 10.1186/s12909-021-02589-9

**Published:** 2021-03-12

**Authors:** Rodrigo Mariño, Julie Satur, Eren Tuncer, Megan Tran, Elizabeth Milford, Vivien Minh Thien Huong Tran, Phuong Qui Tran, Richard Pei-Hua Tsai

**Affiliations:** Melbourne Dental School, University of Melbourne, Melbourne, Australia

**Keywords:** Cultural competence, Dental students, Oral health

## Abstract

**Background:**

Australia possesses a highly multicultural demographic, and thus dental practitioners are likely to regularly encounter culturally and linguistically diverse individuals. It is important for dental practitioners to be culturally competent, however, cultural competency education is highly variable in the curricula of dentistry and oral health courses in Australia, and research is largely limited to dentistry students. This study aims to investigate and compare perceived attitudes, beliefs and practices of cultural competence amongst first and final year Doctor of Dental Surgery (DDS) and Bachelor of Oral Health (BOH) students at the University of Melbourne Dental School.

**Methods:**

Following ethics approval, anonymous questionnaires were completed by 213 participants. The questionnaire was adapted from Schwarz’s Healthcare Provider Cultural Competence Instrument and consisted of five scales. Data was analysed using SPSS V 24.0 software.

**Results:**

A total of 213 students participated in this study (response rate = 88%) The majority of participants were female (*n* = 114, 53.5%) and the mean age of 23.5 years (range 18–40). The majority of participants were Australian born (*n* = 110) with 74.6% (*n* = 159) first generation Australians. Participants who identified as Australian represented 35.7% (*n* = 76) with 66.1% (*n* = 141) identified as partly Australian. Multivariate analysis indicated that, after controlling for other independent variables in the model, those who had the highest cultural competence score were female, who self-identify as “Australian”, who were in the final year. Furthermore, those who were in the final BOH year scored significatively higher than final year DDS students.

**Conclusion:**

The findings of this study suggest that there is a significant difference in students self-reported cultural competence at different stages of their education. This may be attributed to differences in cultural competence education, scope of practice and the type of patient encounters and role modelling that students may experience. Future research should involve follow up to create longitudinal data, as well as research at other dental schools in Australia and overseas.

## Background

An estimated 28.2% of the current Australian population was born overseas. Australia is a federation with six states. Victoria is the second largest state by population (i.e., 6.3 million), and the fastest growing in the nation, with a 1.9% increase in annual migration from overseas [1]. It is well established that cultural minorities and immigrant populations are disproportionately affected by a wide variety of general health and oral health conditions [2]. Therefore, an oral health care professional is highly likely to encounter individuals from a variety of cultural backgrounds on a daily basis during his/her professional life [3–9]. Each culture has unique beliefs, practices, and expectations of oral health care, rendering cultural competence of great importance to dental practitioners.

Underlying cultural beliefs and practices influence the condition of the teeth and mouth, through diet, care-seeking behaviors, or use of home remedies. Individuals from different cultural groups may have oral habits that seem unusual in Western culture. For example, oral hygiene practices (e.g., use of miswak), the habit of chewing betel nut quid, which can damage to the oral mucosa, including the possibility of pre-cancerous or cancerous lesions; tooth lacquering, to name a few [10, 11]. There are also culture influences on expectations, communication and behavior during patient/dental practitioner interactions, which may impact the effectiveness and quality of care.

Therefore, the acquisition and development of cultural competence should begin during a student dental practitioner’s education [8, 12]. With Australia being one of the most culturally diverse countries in the world, it is essential to have culturally competent oral health care professionals in order to address oral health care disparity amongst new immigrant populations as well as supporting interactions with patients from a variety of cultural backgrounds.

Cultural competence amongst health practitioners has been defined in academic literature, as well as in guidelines, policies and standards compiled by the relevant government, health and education bodies worldwide. Betancourt and collaborators [13] define cultural competency in health care as an “understanding [of] the importance of social and cultural influences on patients’ health beliefs and behaviours; considering how these factors interact at multiple levels of the health care delivery system… [and] devising interventions that take these issues into account to assure quality health care delivery to diverse populations.”

Healthcare bodies have adopted such definitions into their relevant policies, albeit in a more concise and simplified form. The Dental Board of Australia’s Code of Conduct outlines the importance of “culturally safe and sensitive practice” [14], acknowledging that differences in the cultural background of both practitioners and patients can influence interactions. Such awareness enables practitioners to adapt appropriately in order to provide good health outcomes [14]. Furthermore, The Australian Dental Council’s (ADC) competency standards used in accreditation of dental education programs require that cultural safety is integrated within the programs to ensure that” students are able to provide culturally safe care for Aboriginal and Torres Strait Islander Peoples.” [15]. This is supported by the University through the valuing of diversity, inclusion and respect for the history and lived experiences and culture of staff, students and the communities they serve [16].

Informed by Betancourt and collaborators’ [13], definition, Schwarz and collaborators [17] describe a model which acknowledges an inherent interaction between cultural awareness and sensitivity, patient-centred communication, and patient centred care orientation, acknowledging the attitudes of health care professionals towards patients and their healthcare experience. In addition, this definition assumes that culturally competent individuals possess knowledge about different languages, beliefs, customs, practices and worldviews.

The teaching of cultural competency skills in dental courses has been assessed. Around the world, dental schools have reported cultural competency as a core component of the dental professional curricula. Some schools have included a devoted subject or have integrated cultural competency into other educational experiences, such as in clinical rotations in North America [18, 19]. In Australia and New Zealand, Nicholson and collaborators [9] survey of various dentistry, dental hygiene and oral health therapy courses across Australia and New Zealand demonstrated that almost all courses incorporated one or more elements.

Strauss and collaborators [20], in North American and Nicholson and collaborators [9] in Australia and New Zealand, observed that dental schools favoured learning techniques such as lectures as the most frequently used teaching method of cultural competency, followed by discussion, self-directed learning, group work and workshops. Mariño and collaborators [21] and Wagner and collaborators [22] also identified that elements of cultural competency were incorporated into other aspects of the dental course curricula, such as clinical placements and work integrated learning. In addition to conventional training, there is evidence that dental students benefit from interactive learning opportunities to effectively improve their self-efficacy and cultural competence, with 80% of students indicating feeling more comfortable working with people of diverse backgrounds following these experiences [23].

Forsyth and collaborators [24] devised a model highlighting the need for cultural competence to be incorporated into university strategic plans at university-wide, faculty, and school levels, to ensure students achieve a minimum level of cultural competency upon graduation. Although this was devised for Indigenous cultural competence in dentistry, the model may apply to general cultural competence for all medicine and health curricula. Attention to this as an important strategic direction for the school, faculty and university has been articulated through both structural and policy approaches in recent years [16, 25, 26].

Clinical supervisors, when surveyed said that the current curricula and examinations of both Doctor of Dental Surgery (DDS) and Bachelor of Oral Health (BOH) students at the University of Melbourne Dental School (UoMDS) did not focus enough on cultural issues and that additional training would be beneficial, especially in areas of patient management and rapport building [19]. Supervisors suggested that cultural competence should be a recurring theme throughout the courses each year, rather than limited to clinical placements. This would enable dental students to communicate more effectively with patients [5]. This view is supported by a recent systematic review finding that a combination of didactic/ online training, community engagement, and reflective writing increased cultural competence of dental students [27].

In order to gain a broader understanding of how dental students’ cultural competency is influenced by educational programs as they progress through their oral health training, this study aims to investigate perceived attitudes, beliefs and practices of cultural competence amongst first and final year DDS and BOH students at UoMDS. This will be explored via the students’ self-perception of their individual cultural competency. These perceptions of cultural competence, for each respective course may reflect how successful the current curricula are in delivering culturally competent education and may inform future improvements to fulfil student needs.

## Methods

Following ethics approval (ID:1749059.2) the study surveyed UoMDS first and final year BOH (BOH1 and BOH3, respectively) students and first and final year DDS (DDS1 and DDS4, respectively) students using anonymous self-completed questionnaires adapted from Schwarz’s Healthcare Provider Cultural Competence Instrument (HPCCI) [14]. Adaptations were introduced to provide an Australian context and a focus on oral healthcare (e.g., “Language barriers are the only difficulties for recent immigrants to *Australia*”; “I find it difficult to manage cultural issues as well as clinical issues when providing *oral* health care”).

The selection of such cohorts allows the possibility to examine the effect of time in the development of cultural competence amongst specific students over time. However, the present approach was one of cross-sectional measurements of different cohorts. The approach allowed sampling to take snapshots in time to review the cultural competency of new (i.e., BOH1 and DDS1 cohorts) and existing students (i.e., BOH3 and DDS4 cohorts). This permitted an assessment of what cultural competencies the students have in their initial year, and then the overall cultural competencies of those who are in their final year of the programmes (which would be influenced by the curriculum as well as possible external factors). Thus, different samples were used each year.

The HPCCI is comprised of five scales: Awareness and sensitivity (10 items), Cultural competence behaviours (11 items), Patient-centred communication (4 items), Practice orientation (10 items), and Self-assessment of cultural competence (8 items). Responses were recorded on a 5-point Likert scale from ‘Strongly agree’ to ‘Strongly disagree’. The questionnaire also sought information about participant demographics and cultural background.

From the HPCCI [15], items were selected and adjusted to be specifically relevant to the dental professions and applicable to students as opposed to qualified professionals. Data were collected using a paper survey distributed to invited student cohorts at the beginning of appropriate class times, which were subsequently collected upon completion by researchers.

There were two versions of the questionnaire. One consisting of 24 questions that was distributed to the first-year cohorts, and another comprised of 43 questions that was distributed to final year cohorts (Table 1). Questions omitted from the first-year students’ survey included all those items enquiring about practice orientation and patient-centred communication, as well as five items about cultural competence behaviours, as it was determined that such questions could not be accurately answered by an individual without clinical experience. Data were collected during the 2018 academic year. The final year cohorts (DDS4 and BOH3) data was collected around April, approximately mid-way through their final academic year. For the DDS1 first-year cohort data was collected in February, but for the BOH1 cohort be data was collected on what it would the end of their first academic year.

**Table 1:** Healthcare Provider Cultural Competence Instrument (HPCCI)

**Awareness and sensitivity** 1. Race is the most important factor in determining a person’s culture. 2. People with a common cultural background think and act alike. 3. Cultural diversity needs to be considered for each individual and group. 4. If I know about a person’s culture, I do not need to consider their personal preferences for health services. 5. Spirituality and religious beliefs are important aspects of many cultural groups. 6. An individual person may identify with more than one cultural group.	
7. Language barriers are the only difficulties for recent immigrants to Australia. 8. I understand that people from different cultures may define the concept of “health care” in different ways. 9. I think that knowing about different cultural groups helps direct my work with individuals and their families. 10. I enjoy working with people who are culturally different from me. **Cultural competence behaviours** 1. When I identify that cultural heritage and needs of individuals are relevant to my work, I seek information about them. 2. I avoid using generalizations to stereotype groups of people. 3. I am aware of potential barriers to oral health care that might be encountered by people of different cultures. 4. I endeavour to remove obstacles to oral health for people of different cultures when I identify such obstacles. 5. I learn from my peers about people with different cultural heritages. 6. Evaluation from my colleagues about how I interact with people of different cultures is not relevant. 7. I take note of cultural factors during patient interactions, such as during history taking. 8. I ask patients and/or their families about their own understanding of health and illness. 9. I ask patients and/or their families to tell me about their expectations for health services. 10. I seek feedback from patients and/or their families about how I relate to their culture. 11. I find ways to adapt my services to my patient’s preferences. **Patient centred communication** 1. When there are a variety of treatment options, how often do you give the patient and their family a choice when making a decision? 2. When there are a variety of treatment options, how often do you give the patient and their family control over decisions about their treatment? 3. How often do you explore with patients, their own preventive approaches to oral health? 4. The use of interpreters aids in interacting with patients and families from diverse cultural backgrounds. **Practice orientation** 1. I understand how to lower communication barriers with patients and their families. 2. I look for nonverbal cues to add to my understanding about my patients and/or their families. 3. I am able to foster a friendly environment with my patients and their families. 4. I attempt to demonstrate a high level of respect for patients and their families. 5. I consistently reflect on my skills as I work with diverse groups of patients and their families. 6. A genuine sense of trust with my patient and their family is important when interacting with my patients. 7. I make every effort to understand the unique circumstances of each patient and their family. 8. I find it hard to ask patients about their cultural backgrounds. 9. I rarely self-assess my abilities when working with patients and their families of diverse backgrounds. 10. I find it difficult to manage cultural issues as well as clinical issues when providing oral health care. **Self-assessment of cultural competence** 1. The oral health practitioner is the one who should decide what is talked about during a visit. 2. The patient and their family should rely on their oral health practitioners’ knowledge and not try to find out about their condition(s) on their own. 3. When oral health practitioners ask a lot of questions about a patient and their family’s background, they are prying too much into personal matters. 4. If oral health practitioners are truly good at diagnosis and treatment, their interactions with patients and their family are not that important. 5. The patient and their family should be treated as if they are partners with the oral health practitioner, equal in power and status. 6. When the patient and their family disagree with their oral health practitioner, it signifies that they do not respect or trust the health care provider. 7. A treatment plan cannot succeed if it is in conflict with the patient and their family’s lifestyle or values. 8. It is not that important to know a patient and their family’s culture and background to treat the client’s illness.	

A power analysis determined that a sample size of 26 participants in each group would yield a power of .80 at a significance level of 0.05 with an estimated effect size between medium and large. Reliability and validity of the revised HPCCI instrument was reviewed. Cronbach’s alpha was used to determine the internal consistency. The reliabilities of the five scales were found to be in the range of 0.79 to 0.99. Construct validity of the scale was assessed through a factor-analysis of the instrument’s 43 items using the maximum-likelihood estimation method with oblique rotation. The analysis indicated that the factor structure of the revised HPCCI approximated the five dimensions of the original instrument [28].

The analysis included the dependent variables represented by the five cultural competence scales scores and seven independent variables. Five cultural competence scores were computed by adding the responses across each of the five cultural competence items. Additionally, in order to quantify all responses, an overall cultural competence index was created by adding the responses across all of the five cultural competence items.

In addition to age, sex, year of study and course of study, and the ethnicity of the student sample was also collected. Ethnic self-identification was operationalized as the way one thinks of oneself [29], and was classified into five groups; “Australian”; “Other: [To be completed], but more Australian”; “Other: [To be completed] & Australian equally”; “Australian, but more Other: [To be completed]”; and “Other: [To be completed]”.

The generation of the student was determined by report of place of birth of the participant, their parents and grandparents. Participants were also asked about the language spoken at home. For those not born in Australia (*n* = 89), the length of time in Australia was also collected.

The statistical analysis provides basic descriptive information on the distribution of the socio-demographic, course and immigration variables. To investigate the patterns of responses for each cultural competence subscale and to determine whether there were similarities between cohorts, Kruskal-Wallis tests were conducted to compare responses for each of the individual items of the five value sub-scales due to the ordinal nature of the data. To test whether first year and final year students had the same pattern of means for each of the cultural competence subscales, Analysis of Variance (ANOVA) were employed. A significant ANOVA was followed by post-hoc comparisons using Tukey’s tests. To test if any combination of the various socio-demographic course and immigration variables (e.g., age, sex, course of study, year, and ethnicity), provided a multivariate explanation of the cultural competence scores, a linear regression model was fitted using a stepwise selection method. When a probability value was 0.05 or smaller, the finding was considered to be statistically significant. Data manipulation and analyses were conducted using SPSS PC (Version 26.0).

## Results

Two-hundred and thirteen students opted to participate in the study out of a possible 242, yielding an overall response rate of 88%. The sample consisted of 27 first year and 25 third (i.e., final) year BOH students and 90 first year and 72 fourth (i.e., final) year DDS students. Participants in all four groups were primarily females. The exception was the DDS final year group, with the proportion of male respondents 54.2%. The average age was 23.5 years (range 18–40 years) One-hundred and sixty-two participants were DDS students (76.1%); and 51 were BOH students (23.9%). One-hundred and sixteen were first year students (54.5%) and 97 were final year students (55.5%). Table 2 presents the distribution of students by year of study and by profession.

**Table 2:** Participants socio-demographic characteristics and mean and standard deviation by course and year of study

Cohort	BOH1 (n-26)	BOH3 (n-25)	DDS1 (n-90)	DDS4 (n-72)	All (n-213)
Mean age (years)	20.9	22.5	22.5	26.1	23.5
Male (%)	23.1	36.0	50.0	54.2	46.5
Australia- born (%)	57.7	72.0	45.6	50.0	51.6
Ethnic identification (%)					
Australian	38.5	52.0	26.7	40.3	35.7
Ethnic group but more Australian	7.7	12.0	11.1	8.3	9.9
Ethnic group Australian equally	15.4	8.0	13.3	5.6	10.3
Ethnic group but less Australian	3.8	20.0	7.8	13.9	10.8
Ethnic group	34.6	8.0	41.1	30.6	32.9

*BOH1* First year Bachelor of Oral Health, *BOH3* Final year Bachelor of Oral Health, *DDS1* First year Doctor of Dental Surgery, *DDS4* Final year Doctor of Dental Surgery

With regard to ethnic background, the largest group self-identified as “Australian” (35.7%), and 31.0% self-identified as a combination of Australian and another ethnicity to some degree (See Table 2). The remaining participants identified solely with ethnic backgrounds other than “Australian” (32.9%). Some participants did not specify their ethnicity (0.4%). Participating students were mainly Australian born (52.6%), of which a majority were first generation (74.6%). The remaining participants were second generation (3.7%) and third generation (21.7%) Australians. Apart from Australia, students named another 26 countries of birth, most frequently mentioned were: Canada (7.4%); China (7.4%); and South Korea (6.3%). Most students indicated speaking English consistently at home (61.5%). Students who did not speak English consistently at home (*n* = 103) indicated speaking 21 other languages, mainly Mandarin (24.4%), Cantonese (11.0%), Korean (12.2%), Arabic (9.7%), or Vietnamese (8.5%).

When comparing first year cohorts, statistically significant differences were found in two of the 24 items: Cultural competence behaviours (“When I identify that cultural heritage and needs of individuals are relevant to my work, I seek information about them“); and Self-assessment of cultural competence (“The patient and their family should rely on their oral health practitioners’ knowledge and not try to find out about their condition(s) on their own“). In contrast, when comparing the two final year cohorts’ responses, statistically significant differences were found in 9 out of 43 items (p<0.05). This included, Awareness and sensitivity (“Cultural diversity needs to be considered for each individual and group”; “Spirituality and religious beliefs are important aspects of many cultural groups”; and “I think that knowing about different cultural groups helps direct my work with individuals and their families”); Cultural competence behaviours: (“I find ways to adapt my services to my patient’s preferences”); Patient centred communication: (“The use of interpreters aids in interacting with patients and families from diverse cultural backgrounds”); Practice orientation: (“I understand how to lower communication barriers with patients and their families”; “I look for nonverbal cues to add to my understanding about my patients and/or their families”; and “A genuine sense of trust with my patient and their family is important when interacting with my patients”); and Self-assessment of cultural competence: (“It is not that important to know a patient and their family’s culture and background to treat the client’s illness”) (Table 1).

As there were a few participants who did not complete the full questionnaire, the analysis that follows only includes those students with no missing answers (*n* = 187; 27 first year and 23 third year BOH students and 89 first year and 48 fourth year DDS students). Means and standard deviation of the cultural competence subscales scores by course of study are presented in Table 3*.* As expected, when first and final year scores were compared, they were statistically significantly different (*p* < 0.001). However, there were not statistically different scores between the first-year student cohorts (i.e., BOH1 compared with DDS1). The exception was self-assessed cultural competence where BOH1 scored higher than DDS1 (1.56 vs. 1.24; *p* < 0.05). On the other hand, when scores from final year DDS were compared to final year BOH, statistically significant differences were found in the responses to the Self-assessed cultural competence (*p* < 0.01); and the Cultural competence behaviours (p < 0.05) subscales (Table 3).

**Table 3:** Participants mean and standard deviation in Cultural Competence scale and subscales by course and year of study

Scale	BOH1 (n-27)	BOH3 (n-23)	DDS1 (n-89)	DDS4 (n-48)
Awareness and sensitivity	8.13 (1.21)	8.13 (1.21)	7.82 (1.64)	7.56 (1.35)
Cultural competence behaviours	4.35 (1.33)	8.17 (2.34)	4.65 (1.28)	6.88 (2.37)
Patient centred communication	–	3.65 (0.57)	–	3.40 (1.01)
Practice orientation	–	6.86 (1.06)	–	6.41 (1.38)
Self-assessed cultural competence	1.56 (0.70)	6.21(1.28)	1.24 (0.71)	5.06 (1.95)
Overall Cultural competence	–	33.04 (3.72)	–	29.31 (5.01)
Overall Cultural competence (adjusted for first years)	13.74 (2.19)	19.22 (2.58)	13.71 (2.65)	16.79 (3.39)

*BOH1* First year Bachelor of Oral Health, *BOH3* Final year Bachelor of Oral Health, *DDS1* First year Doctor of Dental Surgery, *DDS4* Final year Doctor of Dental Surgery

The overall cultural competence scores ranged from 1.0 to 41.0, with a mean value of 20.0 (s.d. 8.9), with significant differences by cohort (*p* < 0.001). While BOH1 and DDS1 students’ scores were not statistically significant [13.7 (s.d. 2.2), and 13.7 (s.d. 2.7), respectively], BOH3 and DDS4 students’ scores [33.0 (s.d. 3.7), and 29.3 (s.d. 5.0), respectively] were statistically significantly, with BOH3 having higher scores than DDS4 (p < 0.01). Additionally, cultural competence scores from first year students were compared to final years’ scores by course with scores corrected for questions omitted. In both courses, scores were statistically significantly higher in the final year compared to first year (13.8 vs. 19.2; *p* < 0.0001 for BOH and 13.7 vs. 16.8; p < 0.001 for DDS).

Statistically significant scores in the overall cultural competence were also found by country of birth, where Australia-born participants scored higher than those born-overseas (21.44 vs. 18.61; *p* < 0.05), and by ethnic self-identification, where those who self-identified as “Australian” had higher scores than those with “Other” self-identifications (21.78 vs. 17.02; p < 0.05). No significant differences were present by age, years in Australia, sex, generation, or language spoken at home.

To better understand the variance in the overall cultural competence score, seven socio-demographic and immigration variables (age, sex, self-reported ethnicity, generation, language spoken at home, country of birth and course-year), were entered into a multiple linear regression model. Three variables (i.e., sex, self-reported ethnicity, course and year of study) remained significant in the final model [F(5,182) = 233.48 *p* < 0.0001]. The resulting model indicated that, after controlling for other independent variables in the model, those who had the highest cultural competence score were female, who self-identified as “Australian”, and who were in the final BOH year. The variance accounted for, using the full model, was 86.3% (adjusted R^2^ = 0.863) (See Table 4).

**Table 4:** Final multivariate model identifying the Cultural competence Score score

Independent variable	Multiple regression coefficient *B* (Std. Error)	*p*
Female (No = 0; Yes = 1)	1.70 (0.50)	0.001
Self-identification (Australia: No = 0; Yes = 1)	1.63 (0.49)	0.001
DDS1 student (No = 0; Yes = 1)	−15.42 (0.60)	0.0001
BOH1 student (No = 0; Yes = 1)	−15.90 (0.81)	0.0001
BOH3 student (No = 0; Yes = 1)	3.43 (0.85)	0.0001
Intercept Adjusted r^2^ = 0.863	27.64 (0.60)	0.0001

## Discussion

Dental students need to acquire the ability to effectively interact with and provide holistic, person-centred care for patients from diverse backgrounds, and also to understand the social, structural and cultural influences on oral health behaviour that are common in a multicultural society such as Australia. They need to develop attitudes that accommodate difference as well as the ability to communicate effectively with patients across cultures. Findings from this study suggest that dentistry and oral health students exhibited dissimilar levels of self-perceived cultural competence at different stages of their education and also within different courses. Overall results indicate that, at the end of their professional training, students’ exposure to professional education and socialization would have resulted in an overall improved score on the cultural competence survey. Australian Dental Council requirements include students’ acquisition of core cultural competency skills to provide culturally safe oral health care, at the end of their professional training [15]. Of course, further assessments of students’ cultural competence need to be conducted using multiple approaches to feel confident that learning outcomes in cultural competence and safety have been met.

Furthermore, multivariate results indicate that final year BOH students scored significantly higher in cultural competence, when compared to final year DDS students. These students were from two different courses, one of which is an undergraduate degree (i.e., BOH) whilst the other is a graduate degree (i.e., DDS). These courses also lead students into different dental professions with a differing scope of practice. Therefore, despite their exposure to patients being largely in the same clinics with patients drawn from the same pools, their clinical experiences between courses varied. In a review of cultural competence content in the DDS and BOH curricula, the DDS course showed less time devoted to formal teaching of this content compared to BOH courses [6, 21].

It has also been identified that cultural competency skills can be developed indirectly via clinical placements and via interactive learning programs [5, 22]. In addition, a detailed assessment of the extent of cultural competence education, which took into consideration all written and current documentations of the curriculum at the UoMDS, as well as the number of formal contact hours and credit points allocated [6, 18], reported that the BOH course assigned fifty-four hours to the teaching of cultural competence content throughout the first 2 years of the course [21]. Whereas the DDS course allocated forty hours confined to the first year of the course [21]. However, that assessment was done just counting documented hours and some other content (such as role modelling, clinical mentoring and student reflective practice) may not be visible in the curriculum of both courses using this method.

Other reports indicated that the teaching techniques used might have been ineffective. Strauss and collaborators [20] observed that most North American dental schools favoured less effective and passive learning techniques such as lectures. This inadequacy was also reported by dental students and graduates who indicated that inclusion of cultural awareness was important in the dental curricula, but that the current curricula needed revising to develop their skills in interacting with patients [7]. In another study, Saleh and collaborators [19] found that the time assigned to complete course tasks and the lack of knowledgeable educators on the topic were major contributing factors. This is further supported by the inconsistency of the cultural competence education between dental curricula of various schools in Australia and New Zealand, thus indicating a need for a standard framework for cultural competency education [9].

Clinical placements offer important opportunities for dental students to develop cultural competence skills first-hand via demonstration of patient management by experienced dental practitioners, having direct interactions with patients and their families, as well as interaction with clinical supervisors that are observing them and are able to give them further insights [5].It has been reported that students interacting with patients from diverse cultural groups and reflecting upon these experiences, assists in refining students’ existing beliefs about particular cultural groups preventing stereotyping [30].

The development of cultural competence and safety relies on good role modelling and attention from supervisors to cultural competence skills development of students, reflective practice and active assessment of knowledge, attitudes and practice skills [27]. Emphasis should also be placed on providing students with opportunities to reflect on their own and others’ cultural values to help them understand the meaning of new experiences and reflect over how these relate to their current experiences [30].

Continuous teaching with a gradual increase in complexity has been considered the most effective method of teaching cultural competence, thus exposure to these concepts in previous undergraduate study may also contribute to differences between the courses [31, 32]. Interestingly, a study investigating social and cultural teaching of medical students concluded that the teaching made little to no difference when students began clinical rotations [33]. The medical students explained that despite an interest in the content, the goal of clinical practice was to treat everyone neutrally, as if they were cultureless [33]. Similarly, clinical supervisors at the UoMDS commented that DDS students appeared to focus on completing treatment rather than building rapport with patients [5]. It was noted that this may be a result of students being graded mostly on their technical skills, rather than their cultural or communication skills [5, 34].

It is also important to consider, in addition to the effects of course curriculum, students’ socio-demographic, immigration and cultural backgrounds, as these factors may affect an individual’s perceived cultural competency level irrespective of their degree of clinical experience. In this regard, it is possible that socio-demographic background has a bearing on an individual’s perceived cultural competence, as these factors influence their beliefs, expectations and interpretation of their own experiences. Such factors include how the participants identified culturally and whether they were linguistically diverse. The participants’ country of birth or family history of immigration, that is, whether they were overseas born or are first, second or third generation Australian, may further influence how they interpret competence and how culturally competent they perceive themselves to be. However, despite the variation in cultural identity among respondents, a consistent pattern of responses across the scales was evident when comparing Australian and overseas born participants. This may be explained by the idea that country of birth alone may not be an accurate descriptor of cultural background, as there are many other factors such as level of acculturation, self-reported ethnicity and languages spoken, life experiences and socio-economic status that contribute to one’s culture [35–37].

The present analysis of the cultural competence scores indicated that those born overseas tended to have lower scores. Moreover, the multivariate analysis indicated that those who self-identified as “Australian”, scored higher than those who did not. These findings may reflect the culturally located nature of the survey, or the socialisation inherent in Australian dental curricula. Furthermore, overseas born students may also not recognise that their ability to traverse their own and Australian cultures represents an element of competence. This also highlights the notion that there might be a distinction between having a culturally and linguistically diverse background (CALD) and being culturally competent. It cannot be assumed that a CALD individual possesses cultural competence because of birthplace, cultural background, or the languages that he or she speaks [38], but rather that this is developed from tolerance, empathy and understanding of cross-cultural issues. This concept was also challenged by Mariño and his collaborators, who found that dentistry students reported cultural values that were largely similar regardless of their backgrounds [39].

Although this study provides valuable insights into the cultural competence acquisition process among dentistry and oral health students, it is not without limitations. The most obvious limitation was the cross-sectional nature of this study, which precludes a strong conclusion about increasing cultural competence with exposure to dental professional education, or the use of year of study as a good proxy for years of exposure to professional socialization. This introduces the influence of variation between cohorts in terms of socio- demographics, personalities, experiences and other factors. As such, it may be that some significant differences have resulted from this. Additionally, the study relied on self-reported data, which may not be an accurate reflection of the relationship between self-perceived and actual culturally competent practice [7, 40]. Another concern is that participants were all students at the UoMDS. As a result, conclusions drawn from this study may not be representative of the cultural competency of all Australian dental profession students, or students at dental schools elsewhere [41]. However, considering these limitations, we believe that the current approach was adequate given the exploratory nature of the study.

## Conclusions

The findings of this study demonstrate that there is a significant difference in self-reported cultural competence as students progressed through their dental training. Students tended to exhibit increasing levels of self-reported cultural competence as they progressed in their studies, but also depending on their course.

Future research should involve the collection of longitudinal data to explore how cultural competence changes over time. Additionally, exploring embedded curriculum content, unconscious bias, reflective practice and role modelling, with explicitly aligned learning objectives and assessments, will enhance future cultural competence curricula in dental and oral health programmes. Collaboration with other dental schools in Australia and overseas would be beneficial in confirming the present results and understanding the influence of cultural competence education on dental students as future dental practitioners.

## Data Availability

Ethics approval was granted on the basis that only researchers involved in the study and could access the de-identified data. The minimum retention period is 5 years from publication. Supporting documents are available upon request to the corresponding author.

## Abbreviations

ADC: Australian Dental Council
ANOVA: Analysis of Variance
BOH: Bachelor of Oral Health
BOH1: First year Bachelor of Oral Health
BOH3: Final year Bachelor of Oral Health
CALD: Culturally and linguistically diverse
DDS: Doctor of Dental Surgery
DDS1: First year Doctor of Dental Surgery
DDS4: Final year Doctor of Dental Surgery
HPCCI: Healthcare Provider Cultural Competence Instrument
UoMDS: University of Melbourne Dental School
